# Learning computer-aided manufacturing from demonstration: a case study with probabilistic movement primitives in robot wood carving

**DOI:** 10.3389/frobt.2025.1569476

**Published:** 2025-05-06

**Authors:** Daniel Schäle, Martin F. Stoelen, Erik Kyrkjebø

**Affiliations:** HVL Robotics, Western Norway University of Applied Sciences, Førde, Norway

**Keywords:** digital fabrication, learning from demonstration (LFD), computer-aided manufacturing (CAM), robot wood carving, probabilistic movement primitives (ProMPs), grasshopper/rhino integration, skill-based toolpath generation, human-robot collaboration in fabrication

## Abstract

Computer-Aided Manufacturing (CAM) tools are a key component in many digital fabrication workflows, translating digital designs into machine instructions to manufacture physical objects. However, conventional CAM tools are tailored for standard manufacturing processes such as milling, turning or laser cutting, and can therefore be a limiting factor - especially for craftspeople and makers who want to employ non-standard, craft-like operations. Formalizing the tacit knowledge behind such operations to incorporate it in new CAM-routines is inherently difficult and often not feasible for the ad hoc incorporation of custom manufacturing operations in a digital fabrication workflow. In this paper, we address this gap by exploring the integration of Learning from Demonstration (LfD) into digital fabrication workflows, allowing makers to establish new manufacturing operations by providing manual demonstrations. To this end, we perform a case study on robot wood carving with hand tools, in which we integrate probabilistic movement primitives (ProMPs) into Rhino’s Grasshopper environment to achieve basic CAM-like functionality. Human demonstrations of different wood carving cuts are recorded via kinesthetic teaching and modeled by a mixture of ProMPs to capture correlations between the toolpath parameters. The ProMP model is then exposed in Grasshopper, where it functions as a translator from drawing input to toolpath output. With our pipeline, makers can create simplified 2D drawings of their carving patterns with common CAD tools and then seamlessly generate skill-informed 6 degree-of-freedom carving toolpaths from them, all in the same familiar CAD environment. We demonstrate our pipeline on multiple wood carving applications and discuss its limitations, including the need for iterative toolpath adjustments to address inaccuracies. Our findings illustrate the potential of LfD in augmenting CAM tools for specialized and highly customized manufacturing tasks. At the same time, the question of how to best represent carving skills for flexible and generalizable toolpath generation remains open and requires further investigation.

## 1 Introduction

Digital fabrication has transformed manufacturing by enabling makers to turn complex digital designs into physical objects, but its potential can sometimes be limited by the rigidity of conventional computer-aided manufacturing (CAM) tools. CAM comprises software tools that translate digital designs (e.g., CAD-models) into machine-readable instructions (e.g., G-Code) for manufacturing, and is an integral part of digital fabrication. Yet, most CAM tools are tailored for standard manufacturing processes such as milling, 3D-printing and laser cutting, where the machines, tools and materials involved, and their interactions, are well understood. Thus, machine instructions can be inferred from geometries based on (formalized) manufacturing expertise and rule-based computational logic that are embedded into CAM software.

For craftspeople and makers that want to employ a non-standard, handcraft-like manufacturing process, existing CAM tools are therefore of little avail. At the same time, writing new CAM routines *ad hoc* is not straightforward, since not all CAM practitioners can program, and it is often tedious and difficult to formalize the tacit knowledge behind such manufacturing processes into a set of rules - even for expert craftspeople it can be challenging to express such knowledge in other means than by doing the actual craft. A way to bypass this difficulty is to record an expert during their manual fabrication process and then make a machine replicate the actions of the maker. Such record-and-play techniques, as discussed in Tian et al. (2019), date back as far as the late 1940s in the context of CNC machining and are still relevant today. For instance, Wölfel et al. (2021) demonstrate how manual fabrication processes can be replicated with a collaborative robot (cobot) using a record-and-play-like approach. Record-and-play drastically simplifies the programming pipeline, even for more intricate operations, and typically results in a repeatable but static fabrication process.

In robotics, Learning from Demonstration (LfD) denotes techniques related to record-and-play, though with an emphasis on generalization, meaning that the goal is to learn a model of a task that can be adapted to variations in the task’s parameters (Billard et al., 2016). The human expert provides multiple demonstrations of a task to generate data that ideally covers most of the variety in the task parameters. A model is trained on the demonstration data to learn relationships between the task parameters during successful executions of the task. During execution, the model is used to generate robot controllers adjusted to the task parameters at hand, and variations in the task can be handled without recording further demonstrations. In this context, the models that are learned from demonstration are often referred to as movement primitives, as they represent a certain motor skill and function as building blocks of motion.

**FIGURE 1:**
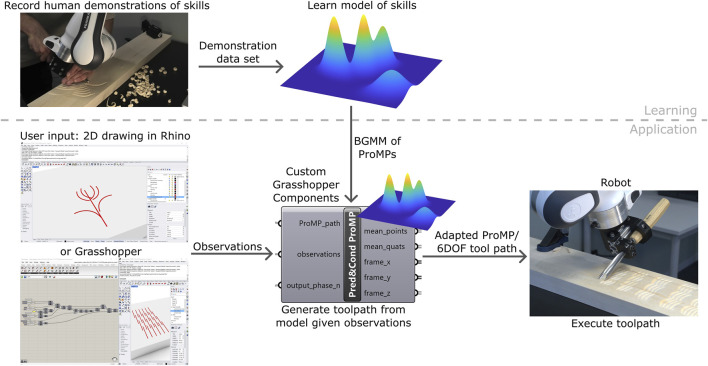
Overview of the pipeline for learning Computer-Aided Manufacturing from demonstration presented in this paper. The pipeline consists of a learning part and an application part. In the learning part, the maker provides demonstrations of manufacturing skills and a probabilistic model representing these skills is learned from the data. The learning part only has to be done once for a set of skills. In the application part, the maker specifies their design input as a 2D drawing made in Rhino with conventional CAD drawing tools for lines and curves, or in Grasshopper as a parametric curve definition, or both. The drawings are discretized and passed as observations to the learned model which is exposed in Grasshopper through custom components. The model uses the observations to adapt the most probable learned skill to the drawings and returns a corresponding 6 degree-of-freedom toolpath that can be executed by the robot.

In this paper, we explore the use of LfD to achieve CAM-like functionality in a digital fabrication workflow for wood carving with hand tools (Figure 1). To this end, we integrate probabilistic movement primitives (ProMPs) (Paraschos et al., 2013) into Grasshopper, which is the node-based programming environment for parametric and algorithmic modeling in Rhinoceros 8 (Rhino) (Robert McNeel and Associates, 2024), a CAD package popular in the maker community.

For wood carving with chisels or gouges, it is not obvious how to compute all the aspects of a toolpath from a digital design, for example, the orientations of the gouge with respect to the wood surface and cutting direction. The non-homogeneous and anisotropic properties of wood also play a role in how a cut is performed in the best way. Thus, we try to capture the essence of, and the tacit knowledge behind, carving motions by observing a human doing sample cuts and learning (a mixture of) ProMPs to capture the joint distribution of the degrees of freedom (DoFs). We then expose the learned ProMPs as custom nodes in Grasshopper, where they can be referenced to user-created geometries, and output the according carving toolpath that can be simulated and saved to a file to be executed by a robot. Our approach allows the maker to both specify designs and generate toolpaths based on human craft skills in the same familiar CAD environment. We evaluate our approach on four basic wood carving applications, and investigate in more detail the effect of two types of toolpath adjustments that were required to achieve the presented results.

## 2 Related work

Leveraging human craft skills to inform machine-driven manufacturing processes has been explored before in both research and industry. The various ways and applications in which the topic has been approached highlight both its potential for diverse manufacturing scenarios and the ambiguity surrounding the best methods to transfer tacit knowledge from humans to machines.

The approaches in the literature can be broadly categorized into three main groups: 1. Manual analysis of human demonstrations to identify characteristic rules and parameters, which are then incorporated into machine instructions. 2. Record-and-play techniques, which take a single human demonstration, perhaps apply some manual or automatic processing to optimize and adapt the toolpath to the embodiment of the machine, and then replay the demonstration as accurately as possible. 3. LfD techniques, which use multiple human demonstrations to learn a generalized model of the operation. This model can then be used to generate toolpaths based on specific task parameters.

An example of the first group is presented in Steinhagen et al. (2016), which aims to recreate traditional stone surface structuring with an industrial robot. The authors record chisel poses and hammer velocities with a high speed camera during demonstrations of different surface structuring techniques performed by expert craftspeople. Instead of using these recordings as training data in a machine learning based approach, they analyze the different techniques together with the expert craftspeople and try to identify the most important process parameters manually. The identified parameters are then exposed in a custom toolpath programming interface such that the maker can easily experiment with the effect of these parameters and iterate on their design. In a more recent paper, Shaked et al. (2021) follow a similar approach. The authors analyze stone surface structuring techniques of craftspeople in a motion capturing setup, tracking the chisel pose during sessions consisting of multiple strokes. They analyze the chisel movement and manually extract process parameters that characterize three different techniques. Informed by the extracted parameters, a parametric toolpath consisting of multiple strokes is created manually in Rhino/Grasshopper and executed by an industrial robot fitted with a pneumatically actuated stone carving tool. The resulting stone surface is captured with a 3D camera and imported back into Rhino in order to adapt the subsequent processing passes to the new surface structure. How exactly these adaptations are done remains unclear from the paper. Ma et al. (2021) adopt a comparable strategy for robotic clay sculpting. The authors also identify process parameters that have a strong influence on the aesthetic quality of the physical result and expose these parameters to the maker in a custom user interface. The computation of the toolpath is then treated as an optimization problem, where the desired geometry and the chosen process parameters are taken into account.

An example for the second group, record-and-play techniques, is the work by Wölfel et al. (2021). The authors propose a system for reproducing handcraft with a cobot to enable makers to mass produce objects they created with hand-held tools. The system consists of a marker-based motion capture setup to record the motion of the tool while the maker produces a workpiece manually, a graphical user interface that remaps the recorded motions to robot configurations and allows the user to simulate, modify and verify the toolpath, and a KUKA LWR IV collaborative robot to reproduce the operation. To modify the toolpath, the user can click-and-drag waypoints in the simulated robot environment. The replicated handicraft operations presented in the paper are drawing/painting on 2D- and 3D-surfaces and carving in soft modeling clay. Tian et al. (2019) explore new interactive fabrication patterns by leveraging shared machine control and computationally defined haptic feedback. Their work can be considered as an example for the second group, as the presented custom desktop lathe is able to record and replay all manual control inputs. However, the transfer of tacit knowledge from man to machine is not the main focus of the paper.

The third group are LfD-related techniques. LfD is a wide-spread technique in robotics and robot learning, but has seen less application in the context of digital fabrication and CAM. Nevertheless, some relevant examples of the third group can be found in the literature. For instance, Park et al. (2022) explore the use of LfD to learn brush strokes for digital painting, with a focus on reducing the gap between simulated and real-world robotic painting. To this end, they collect human demonstrations of brush strokes and the corresponding painting outcomes on a digital canvas in a teleoperation setup. This dataset is used to train a deep latent variable model that encodes the joint distribution of low-level robot controls and painting outcomes. The learned model is then used to infer robot controls given a target image. The target image is recreated on a digital canvas by executing the control commands on a 4DoF robot featuring a digital stylus and force control.

Another example is found in Rossi and Nicholas (2019), which investigate the use of convolutional neural networks (CNN) as a design tool for forming sheet metal with an English Wheel operated by an industrial robot. They use the CNN to learn a mapping between the tracking pattern of the wheel on the sheet metal surface and the resulting double curved topology of the sheet metal. As training data, not a human craftsperson is observed, but manually generated tracking patterns are executed with the robot. The resulting sheet metal topologies are recorded with a 3D scanner. The trained network can then be used to infer the tracking pattern, a fundamental aspect of the toolpath, based on the topology of the digital model of the sheet metal. However, it remains unclear from the paper how the full 6DoF toolpath required to control the robots end effector is computed from the tracking pattern.

Highly relevant to this paper is the work by Brugnaro and Hanna (2017), which introduces an LfD approach to train an industrial robot to do wood carving using traditional gouges, though assisted by an electric reciprocating tool. The reciprocating tool generates a small amplitude, high frequency movement of the blade along the tool axis which reduces the force necessary to cut through the wood. The authors argue that such reciprocating tools are commonly used by professional craftspeople and do therefore not alter the way in which craftspeople use traditional carving tools. The authors use a neural net as a regression model to capture the relationship between parameters characterizing toolpaths and parameters describing carved geometry. The parameters characterizing a toolpath are the feed rate, the tool angle with respect to the wood surface and grain direction, as well as the cutting force estimated from the current drawn by the reciprocating tool. The parameters describing the geometry of the resulting cut are the cut length and depth profile. The training data for the neural network is collected in two stages. At first, a series of human demonstrations of straight cuts is recorded in a motion capture setup and the network is trained on the extracted parameters. The resulting network is then used to generate toolpaths that make up a second, bigger data set consisting of interpolations between the toolpath parameters found in the human demonstrations. The toolpaths in the second data set are executed by the robot without human intervention, the carving results captured with a 3D camera and a new network is trained with the extracted parameters. This network is used to predict the tool angle with respect to the wood surface and the depth profile of a cut, given a straight reference toolpath of a certain length. Real-world usage of the network is demonstrated by carving three circular patterns consisting of such straight cuts into lime wood with the robot. However, it remains unclear from the article how exactly these patterns were composed in the digital design space to achieve apparently curved depth profiles and material removal that exceeds the depth of single cuts. By including the cutting force as well as the tool angle with respect to the grain direction in the training data, the system can take into account some of the anisotropic properties of wood. But if and how force feedback and the grain direction were utilized during the robotic carving of the circular patterns is not apparent from the article.

A direct example of force feedback integration in robot wood carving is the dynamic toolpath adaptation scheme proposed by Nakamura and Hirasawa (2021), though it does not follow an LfD approach. The authors manually generate an initial toolpath, which is modified during execution when the load on the tool exceeds a threshold. To this end, they use a force sensor between the tool and the robot flange to measure the cutting force along the tool axis. When the threshold is exceeded, an adaptation routine is triggered and the toolpath is re-planned to first relieve the stress on the tool by moving backwards, and then to continue a shallower cut by moving the toolpath closer to the wood surface.

Contrary to Wölfel et al. (2021), which focuses on replicating specific manufacturing operations for mass production, we aim to learn more generalized manufacturing skills, giving the maker greater freedom in the digital design space and a more CAM-like user experience. Since we want to avoid the manual analysis of carving skills we chose to adopt an LfD approach. While Brugnaro and Hanna (2017) learn a relationship between toolpath parameters and certain parameters characterizing the geometry of straight cuts, we choose a purely trajectory-based modeling approach for this initial study, as it allows us to demonstrate arbitrarily shaped cuts to the robot without needing to first identify a suitable method for parametrizing the geometries of such cuts. Moreover, the model naturally represents the temporal correlations and dynamics, which can be relevant properties of carving motions.

## 3 Materials and methods

The materials and methods section is divided into five subsections: First, we outline our robot setup for wood carving. Second, we review the mixture model formulation of ProMPs that we use to represent wood carving skills. Third, we detail our data preparation approach to learn carving skills in local coordinate frames. Fourth, we describe how we use the ProMP model to retrieve 6 DoF toolpaths from two-dimensional input drawings with the proposed integration of ProMPs into the CAD software Rhino. And fifth, we provide additional details on our software implementation.

### 3.1 Set-up and hardware

**FIGURE 2:**
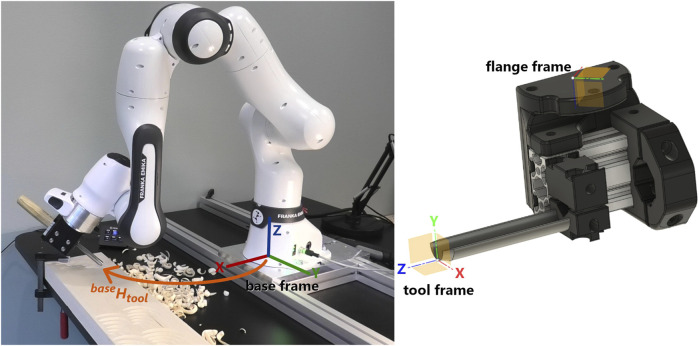
**Left**: Wood carving setup with Franka Emika FR3. **Right**: Custom made fixture for a Pfeil 9/15 gouge. The tool is only hinted at in the CAD model.

In this paper we use a Franka Emika FR3 collaborative robot arm for learning and reproducing wood carving. The use of a collaborative robot allows us to record demonstrations via kinesthetic teaching, which means the operator provides demonstrations with the gouge while it is mounted to the robot. A traditional handheld gouge (Pfeil 9/15) is mounted to the robot’s flange with a custom-made fixture (Figure 2). The tool center point is calibrated to the center of the cutting edge.

The robot is controlled with an task space impedance controller. During kinesthetic teaching, we set the rotational and translational stiffness gains to zero, which renders the robot compliant while compensating for gravity. When the robot is carving wood, we in increase the stiffness gains to $4000\frac{N}{m}$ and $50\frac{N}{rad}$ for translational and rotational DoFs, respectively, which are the highest values we could set before the controller became unstable.

### 3.2 Bayesian Gaussian mixture model of probabilistic movement primitives

For the representation of wood carving skills learned from demonstrations, various models can be considered. Some of the related papers discussed in Section 2 use representations based on neural networks. Also different movement primitive formulations such as Dynamic Movement Primitives (DMPs) (Ijspeert et al., 2013), Task-Parametrized Gaussian Mixture Models (TP-GMMs) (Calinon, 2016) and Kernelized Movement Primitives (KMPs) (Huang et al., 2019) are viable alternatives to the ProMPs chosen in this paper.

Recent adaptations of DMPs (Vedove et al., 2025) appear to be a promising approach to representing manufacturing tasks that involve in-contact movements with complexly shaped workpieces, such as those required for wood carving. TP-GMMs and KMPs allow for learning of skills in local coordinate frames, a desirable property for our application. Additionally, KMPs have shown good generalization capabilities without requiring large amounts of demonstrations (Huang et al., 2019).

In this paper, we use ProMPs to represent the wood carving skills learned from demonstrations, as they provide the best balance in integrating three key properties critical to our work: 1) ProMPs conveniently facilitate the learning of skill libraries. 2) ProMPs have shown good performance in terms of skill recognition and adaptation to multiple via-points (Maeda et al., 2014; Maeda et al., 2017). 3) Recognition and adaptation of ProMPs have simple closed-form solutions. The last point is especially important in consideration of our desired Grasshopper integration. In Grasshopper, each time a parameter or referenced geometry is modified, the whole program is re-executed to give the user immediate feedback of how the change in the input data affects the output. To maintain this swift and reactive user experience also when using skill models to create toolpaths, the adaptation of the skills to drawing inputs by the user must be fast.

A ProMP represents a distribution over trajectories learned from a set of N demonstrations (Paraschos et al., 2013). In this paper, a trajectory $D={\left\{y_{t}\right\}}_{t=1}^{T}$ is a time-series of state vectors $y_{t}$ of dimension D=6 , as defined later in Equation 1. For a more concise representation, trajectories are approximated by a linear regression model

$$y_{t}=\Phi_{t}w+\epsilon_{y}.$$

A weight vector $w\in R^{LD}$ is related at time t to the state $y_{t}$ through a time dependent, block diagonal basis function matrix $\Phi_{t}\in R^{D\times LD}$ . The matrix $\Phi_{t}$

$$\Phi_{t}=\left[\begin{array}{ccc} \phi_{1,t}^{⊺} & \cdots & 0\\ \vdots & \ddots & \vdots \\ 0 & \cdots & \phi_{D,t}^{⊺} \end{array}\right]$$

contains on its diagonal a row vector $\phi_{d,t}^{⊺}\in R^{L}$ for each DoF, which again contains the values of L normalized, evenly spaced, Gaussian basis functions $\phi_{l}\left(t\right)$ evaluated at time t . The weight vector w is a vertical concatenation of D column vectors $w_{d}\in R^{L}$ , representing the weight vectors of each individual degree of freedom of the state. The last term $\epsilon_{y}\in R^{D}$ is a vector containing the observation noise which is assumed to be independent and identically distributed and to follow the normal distribution $N\left(0,\Sigma_{y}\right)$ . The weight vectors ${\left\{w_{n}\right\}}_{n=1}^{N}$ representing the N demonstrations are computed with ridge regression.

$$w_{n}={\left(\Phi^{⊺}\Phi +\lambda I\right)}^{-1}\Phi^{⊺}\tau_{n}.$$

Similar to Kulak et al. (2021), we model the distribution of the demonstrations by learning a Bayesian Gaussian mixture model (BGMM) from the weight vectors. An advantage of the BGMM over a conventional GMM is that it effectively chooses the number of mixture components in the model by assigning mixture weights close to zero to unnecessary components that would result in over-fitting of the data. A BGMM is a mixture of K multivariate normal distributions (MVN).

$$p\left(w|\pi ,\mu ,\Sigma \right)=\overset{K}{\underset{k=1}{\sum }}\pi_{k}\;N\left(w|\mu_{k},\Sigma_{k}\right)$$

where, in respective order, $\pi ={\left\{\pi_{k}\right\}}_{k=1}^{K},\mu ={\left\{\mu_{k}\right\}}_{k=1}^{K},\Sigma ={\left\{\Sigma_{k}\right\}}_{k=1}^{K}$ are the mixture coefficients, mean vectors and covariance matrices parametrizing the distribution. Uncertainty about these parameters is expressed through a Normal-Inverse Wishart prior on the mean μ and covariance Σ

$$p\left(\mu ,\Sigma \right)=\overset{K}{\underset{k=1}{\prod }}N\left(\mu_{k}|m_{0},\frac{1}{\beta_{0}}\Sigma_{k}\right)\text{IW}\left(\Sigma_{k}|S_{0},\nu_{0}\right),$$

and a Dirichlet process prior on the mixing coefficients π .

We will not detail the estimation process of the parameters in this paper. We implemented all required ProMP functionality as Python classes, and use the default BGMM implementation in scikit-learn (Pedregosa et al., 2011) and refer the interested reader to documentation and [Bishop (2006), Ch. 10.2]. The Python classes are later used in script components in Grasshopper.

### 3.3 Data preparation for learning in local frames

Often when learning movement primitives from end effector trajectories, the end effector poses are represented in the robot’s base frame - which makes sense for movements that are somewhat concentrated within a region of the workspace. Of course, it is possible to record demonstrations in different locations and later use conditioning to adapt the movement primitive to the location required for the imminent execution, but it requires more demonstrations to learn primitives that can generalize over a wide range of locations.

In this paper, we want to draw patterns composed of movement primitives at any location and orientation in the horizontal drawing plane in Rhino and execute the patterns somewhere in the robot’s work space, and we assume no correlation between the pose of the cut and the way the cut is performed. Thus, we learn the primitives in local frames instead of the robot’s base frame, enabling us to align, learn, recognize and execute primitives anywhere in the dexterous workspace of the robot arm. The acquired demonstration data consists of homogeneous transformation matrices describing end effector poses in the robot’s base frame (Figure 2) at each time step t

Htool,tbaset=1T∈SE(3).

We exploit common characteristics of all the cutting demonstrations to compute a local frame for each demonstration. From the recording process, we know that in all demonstrations the tool penetrates the surface of the wood at some point, and after that, is laterally constrained by cutting through the wood such that we can expect displacement in the direction of the tool axis only. We also know the height of the wood surface in the robot’s base frame from measuring it with a caliper towards a common reference plane (e.g., table where robot is mounted on to), and we can assume that the wood surface is plane and parallel to the reference plane due to jointing and planing when preparing the wood. Given these characteristics, we use the following heuristic to compute a local frame for each demonstration:

1. Detect the time step s at which the trajectory crosses the wood surface plane and remains beneath it for at least the duration of a window $w_{\mathrm{avg}}$ . The position coordinates at this time step mark the start of the cut and will be the origin of the local frame $O_{\mathrm{local}}$ . The time step e and the position at the end of the cut is computed equally, but looking for a crossing of the surface plane in inverted direction.
2. Compute the y-axis of the local frame $Y_{\mathrm{local}}$ by computing the average of the unit vectors pointing from $O_{\mathrm{local}}$ to the samples in the $w_{\mathrm{avg}}$ and projecting it to the xy-plane of the robot base frame.
3. Define the z-axis of the local frame $Z_{\mathrm{local}}$ to be parallel to the z-axis of the robot base frame.
4. Compute the x-axis of the local frame $X_{\mathrm{local}}$ as the cross product of $Y_{\mathrm{local}}$ and $Z_{\mathrm{local}}$ .
5. Define homogeneous transformation matrix from robot base frame to local frame Hlocalbase from $O_{\mathrm{local}}$ , $X_{\mathrm{local}}$ , $Y_{\mathrm{local}}$ and $Z_{\mathrm{local}}$ .

**FIGURE 3:**
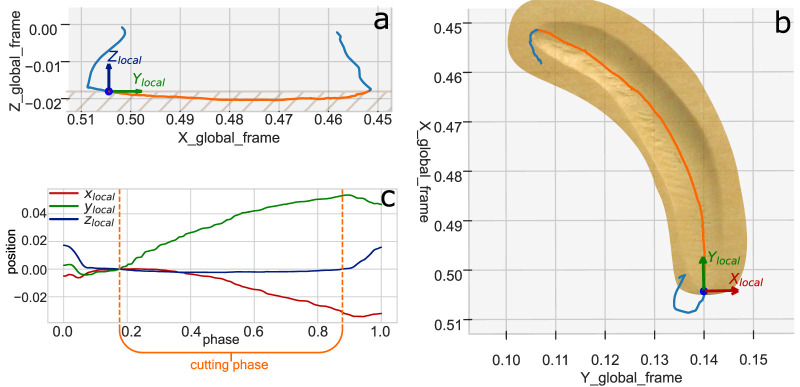
Example of local frame computed for a demonstration of a counterclockwise arc cut. All distances are in meters. **(a)** shows a side view of the recorded toolpath together with its local frame. The blue parts represent approach and retract, the orange part cutting. The hatched region indicates the wood. **(b)** shows a top view of the toolpath over a photo of the carved wood. **(c)** shows the position coordinates of the toolpath in its local frame plotted over the phase (normalized time). Cutting happens between the dashed orange lines.

An example of a demonstration and its local frame plotted in the robot base frame are shown in Figures 3a, b.

Once the local frame is computed for a demonstration, the demonstration can be expressed with respect to the local frame at time step t with

Htool,tlocal=Hlocal−1baseHtool,tbase∀t

With all demonstrations represented in local frames, we can align them temporally based on the start and end of cuts. Our alignment process is as follows:

1. Trim off samples where the tool stands still at the start and end of each demonstration. Account for the trimming in s and e , the start and end index of the cut.
2. Compute a phase signal $z={\left\{z_{t}\right\}}_{t=0}^{T}$ for each demonstration by normalizing its time signal by the last time value.
3. Retrieve the phase values $z_{s}$ and $z_{e}$ at start and end index of the cut for all demonstrations.
4. Compute the mean phase values $\mu_{z_{s}}$ and $\mu_{z_{e}}$ for start and end of the cut across all demonstrations
5. Do a linear interpolation to map the original time signal of the demonstrations to a phase signal, while ensuring that the cut segments align with the mean phase values $\mu_{z_{s}}$ and $\mu_{z_{e}}$ at the cut start and end times.
6. Resample the demonstration such that the previously computed phase signal is evenly spaced between 0 and 1 with a given number of samples.

We have now obtained phase-aligned, locally represented demonstrations that we use as training data for the ProMP model.

To encode the orientations of the end effector in our ProMP model, we first compute the equivalent unit quaternions $q_{t}\in S^{3}$ , with $S^{3}$ denoting a unit sphere in $R^{4}$ , from {Htool,tlocal}t=0T . Then, we compute rotation vectors $\theta u\in R^{3}$ , vectors parallel to the axis of rotation whose lengths are the rotation angles θ , by applying the quaternion logarithm at each time step.

$${\left(\theta u\right)}_{t}=\log\left(q_{t}\right)=\frac{v_{t}}{\left\|v_{t}\right\|}\arccos\left(s_{t}\right)\;\forall t.$$

v is the vector part and s the scalar part of the quaternion. Before computing the logarithm, we ensure that all unit quaternions lie in the positive hemisphere of $S^{3}$ . Finally, the state vector $y_{t}$ which we use in our ProMP model is composed of the positions and rotation vectors, leading to demonstrations D of the form

$$D={\left\{y_{t}\right\}}_{t=1}^{T}={\left\{p_{t},{\left(\theta u\right)}_{t}\right\}}_{t=1}^{T}.$$ (1)

Unit quaternions can be retrieved from the rotation vectors by applying the quaternion exponential

$$\exp\left({\left(\theta u\right)}_{t}\right)=q_{t}=s_{t}+v_{t}=\cos\left(\left\|v_{t}\right\|\right)+\frac{v_{t}}{\left\|v\right\|}\sin\left(\left\|v_{t}\right\|\right)\;\forall t.$$ (2)

### 3.4 Inferring toolpaths from CAD drawings

**FIGURE 4:**
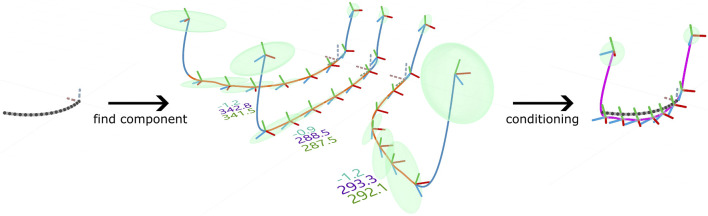
**Left**: An arc shaped cut is specified by a line arc drawn by the user in Rhino. The black dots indicate the observations O extracted from the line. The dashed coordinate frame is the local frame of the observations. **Middle**: Mean trajectories of a BGMM with K=3 components. The blue sections indicate approach and retract and the orange sections cutting. The green ellipsoids represent one standard deviation of the positions. The blue numbers are the log prior probabilities log⁡p(k) of each component, the purple numbers are the log Gaussian likelihoods log⁡p(O|k) of the observations and the green numbers are (proportional to) the log posterior probability of the components given the observations log⁡p(k|O) . **Right**: The most probable mixture component is adapted to the observations by conditioning.

Once we have learned a BGMM of ProMPs to capture the relations of the DoFs during different carving motions, we want to use the model to generate 6DoF Cartesian toolpaths from two-dimensional CAD drawings. We follow a similar approach as Ewerton et al. (2015) used to infer robot trajectories from human motion input during human-robot interaction. This approach has two steps: First, given sparse observations of a subset of the modeled DoFs, determine which mixture component (MVN) explains the observations best. Second, condition the previously identified MVN on the observations to adapt the full movement, including the unobserved DoFs, to the observations. In Ewerton et al. (2015), the observations are Cartesian wrist positions of a human coworker recorded with a motion capturing system, and the unobserved DoFs are the joint positions of a 7DoF robot arm. In our case, the observations are the Cartesian x- and y-coordinates sampled from drawings in the horizontal drawing plane in Rhino. The unobserved DoFs are the remaining coordinates describing the pose of the tool frame: Cartesian z-coordinate and orientation. The process is illustrated in Figure 4.

For the conditioning on CAD drawings, the sketch lines in Rhino are first discretized into m samples connected by equal length segments. A corresponding phase signal $z_{O}={\left\{z_{O,t}\right\}}_{t=0}^{T}$ evolving from $z_{O,0}=0$ to $z_{O,T}=1$ in T=m+1 time steps is computed. With the xy-coordinates of the samples, a local frame is computed for each line. The procedure is similar to that described in Section 3.3, with the difference that $O_{\mathrm{local}}$ is always placed at the beginning of the line, since we only draw the part of the toolpath where the robot will actually carve and thus, start and end index of the cut align with the start and end point of the line. Typically, $w_{\mathrm{avg}}$ is shorter for CAD-drawn lines, since they are sampled more coarsely than the demonstrations.

The state vector representing the CAD lines merely contains the position coordinates along x- and y-axis of the local frame leading to observations of the form

$$O={\left\{p_{t}^{x},p_{t}^{y},0,0,0,0\right\}}_{t}^{T}.$$

To make predictions with our model based on these observations, we have to modify the basis function matrix $\Phi_{t}$ such that it only contains basis functions for the first two, observed DoFs. The basis functions for all other DoFs that are not extracted from the CAD drawings are replaced by zeros, resulting in new basis function matrix ${\hat{\Phi }}_{t}$



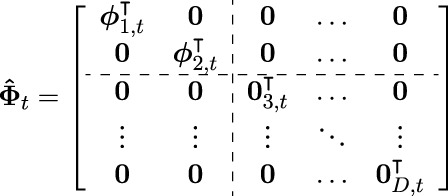




By using ${\hat{\Phi }}_{t}$ , we exclude the inputs from all DoFs except the first two. This allows us to recognize and adapt mixture components based solely on the first two DoFs. However, all DoFs are still indirectly adapted due to the correlations present in the covariance matrices.

To identify the most probable mixture component $k^{*}$ given observations O , we compute the posterior probability p(k|O) for each component and then find the component that maximizes this probability. Formally.

$$p\left(k|O\right)\propto p\left(O|k\right)p\left(k\right)$$ (3)

$$k^{*}=\arg\underset{k}{\max}p\left(k|O\right).$$

In Equation 3, p(k) is the prior probability or mixing weight of component k , and p(O|k) is the Gaussian likelihood of the observations given component k and is computed by

$$\begin{array}{cc} p\left(O|k\right) & =\int p\left(O|w\right)p\left(w|k\right)dw\\ & =\int N\left(w|\mu_{k},\Sigma_{k}\right)\overset{T}{\underset{t=1}{\prod }}N\left(O_{t}|{\hat{\Phi }}_{t}w,\Sigma_{y}\right)dw\\ & =\overset{T}{\underset{t=1}{\prod }}N\left(O_{t}|{\hat{\Phi }}_{t}\mu_{k},{\hat{\Phi }}_{t}\Sigma_{k}{\hat{\Phi }}_{t}^{⊺}+\Sigma_{y}\right). \end{array}$$ (4)

To evaluate the likelihood of the observations at the correct phase values, we embed the phase of the observations $z_{O}$ (encompassing only the actual cut) into the phase signal of the model components z (encompassing approach, cut and retract) such that $z_{O}$ aligns with the cutting segment of the model components. This is done by linear interpolation.

$${\hat{z}}_{O}=z_{s_{k}}+z_{O}\left(z_{e_{k}}-z_{s_{k}}\right).$$

The phase values $z_{s_{k}}$ and $z_{e_{k}}$ at the start and end of cut of the mean trajectories of the K MVNs in the BGMM are computed similarly as described in Section 3.3. Thus, ${\hat{\Phi }}_{t}$ in Equation 4 means we evaluate the basis function matrix at the t -th time step of the phase ${\hat{z}}_{O}$ computed for component k . The same phase signals are also used in the following conditioning step (Equations 5–8).

Once we have obtained $k^{*}$ , we can adapt this component $N\left(w|\mu_{k^{*}},\Sigma_{k^{*}}\right)$ to the observations. To this end, we compute the posterior distribution of w conditional on the observations O with recursive updates for each time step. $O_{t}$

$$p\left(w|k^{*},O\right)=N\left(w|\mu_{k^{*}}^{+},\Sigma_{k^{*}}^{+}\right)$$ (5)

$$\mu_{k^{*}}^{+}=\mu_{k^{*}}+K\left(O_{t}-{\hat{\Phi }}_{t}^{⊺}\mu_{k^{*}}\right)$$ (6)

$$\Sigma_{k^{*}}^{+}=\Sigma_{k^{*}}-K\left({\hat{\Phi }}_{t}^{⊺}\Sigma_{k^{*}}\right)$$ (7)

$$K=\Sigma_{k^{*}}{\hat{\Phi }}_{t}^{⊺}{\left(\Sigma_{y}+{\hat{\Phi }}_{t}^{⊺}\Sigma_{k^{*}}{\hat{\Phi }}_{t}\right)}^{-1},$$ (8)

where the posterior parameters $\mu_{k^{*}}^{+},\Sigma_{k^{*}}^{+}$ computed at time step $O_{t}$ serve as the prior parameters $\mu_{k^{*}},\Sigma_{k^{*}}$ for the update at $O_{t+1}$ . The full toolpath adapted to the CAD drawings is then obtained by computing the conditional probability distribution over the trajectories D given the observations O from the CAD drawings

$$p\left(D|O\right)=\int p\left(D|w\right)p\left(w|k^{*},O\right)dw$$

Which can be evaluated at any time $z_{t}\in \left[0,1\right]$ with

$$p\left(y_{t}|O\right)=N\left(\Phi_{t}\mu_{k^{*}}^{+},\Phi_{t}\Sigma_{k^{*}}^{+}\Phi_{t}^{⊺}+\Sigma_{y}\right).$$

Prior to execution on the robot, the rotation vectors in D are converted into unit quaternions using Equation 2.

### 3.5 Implementation in rhino and grasshopper

**FIGURE 5:**
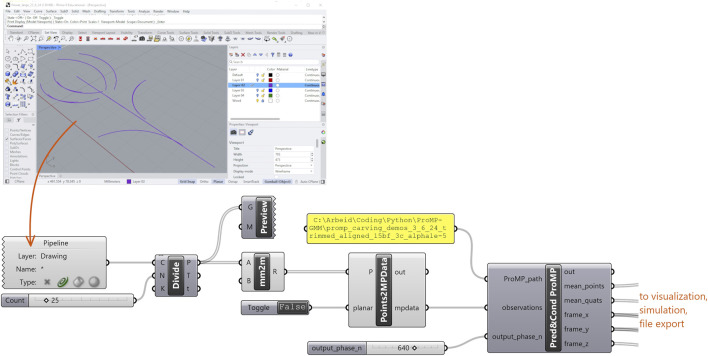
Geometries are sampled from Rhino, divided into n observation samples, the local frames are then computed for the observations, and the observations are passed to a ProMP model for predicting and adapting the most probable mixture component. The mean trajectory is returned, used for plotting, simulation and controlling the robot.

In this paper we use the CAD software Rhino due to its popularity in the digital fabrication community and its integrated visual programming environment Grasshopper. In Grasshopper, we can use scripting nodes to run Python code that interacts with geometries in Rhino. As shown in Figure 5, we use two custom script components to infer toolpaths from drawings as described in Section 3.4: One to transform the observations to local frames and convert them from Rhino point format into our own data type. And a second one that loads a trained BGMM of ProMPs, takes the observations and predicts and conditions mixture components of the BGMM.The resulting toolpath can then be used for simulation within Grasshopper via the “Robots” plug-in (Soler, 2023), which allows us to ensure that the toolpath is collision-free and the robot stays within its joint limits. For controlling the real robot, we save the toolpath as a text file which we then use as reference values for the Cartesian impedance controller. Note that alternatively to exporting an explicit toolpath, the distribution parameters of the adapted mixture component could be exported and used in the robot control loop to evaluate the reference values online based on the current sample time.

The sketching environment in Rhino can be connected continuously to Grasshopper with a “geometry pipeline” which samples Rhino for new geometries and makes them available as a list in Grasshopper. All Grasshopper components process each geometry in the list and output their results as corresponding lists. Any change in the geometry pipeline or any other parameter in Grasshopper triggers a re-computation of the components. This makes for a short iterative loop from design input to toolpath output and enables the user to quickly prototype and experiment with skills learned from human demonstration in a CAD environment. The toolpaths returned for each individual geometry are with travel movements generated in Grasshopper: The end and start of subsequent individual toolpaths are connected with Bezier spans, and the orientations are blended smoothly with spherical linear interpolation (SLERP). One option to sampling the drawings from Rhino is to generate them programmatically in Grasshopper. This gives the user less of a “manual drawing experience” but enhanced parametric control over the geometries.

The integration of ProMPs into Grasshopper leads to what can be seen as a hybrid programming interface that allows the user to combine precise and parametric CAD tools with complex, human-like skills.

## 4 Results

We conducted two types of experiments using the methodology presented in this paper. We first show different carving results to illustrate the typical design workflow, and point out limitations of our approach. And then we provide further details on the effect of two types of toolpath adjustments we used to achieve the previous results.

### 4.1 Data acquisition

**FIGURE 6:**
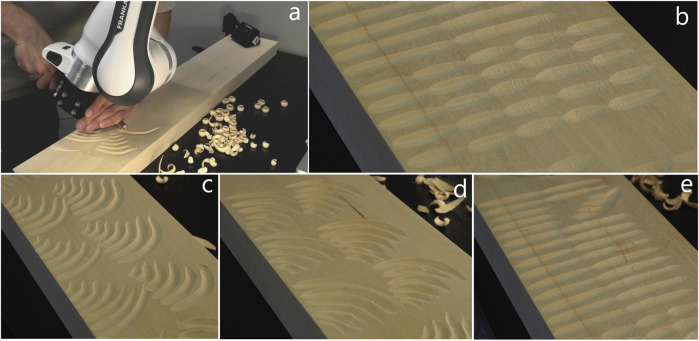
Recording wood carving demonstrations. **(a)** Recording woodcarving demonstrations with kinesthetic teaching **(b)** 25 short straight cuts **(c)** 39 counterclockwise arcs with different radii. **(d)** 35 clockwise arcs with different radii. **(e)** 25 long straight cuts.

For the experiments in this paper we recorded 129 human demonstrations of different wood carving cuts in limewood: 25 short (approx. 30 mm long) straight cuts, 25 long (approx. 60 mm long) straight cuts, 39 counterclockwise arcs and 35 clockwise arcs (approx. 90° with radii from 15 to 60 mm). The demonstrations were performed by a hobbyist wood carver and recorded with kinesthetic teaching (Figure 6). In addition to the trajectories, we recorded point clouds of the demonstrated cuts with a Zivid One+ 3D-camera. A video of the demonstration recording process can be found in the Supplementary Materials (Supplementary Video 1).

### 4.2 Wood carving applications

We have chosen wood carving applications that involve different types of cuts learned from demonstration and different digital design workflows in Grasshopper and Rhino. These are two versions of “Naguri,” a rope shape, and a flower shape, and are described below.

#### 4.2.1 Naguri

**FIGURE 7:**
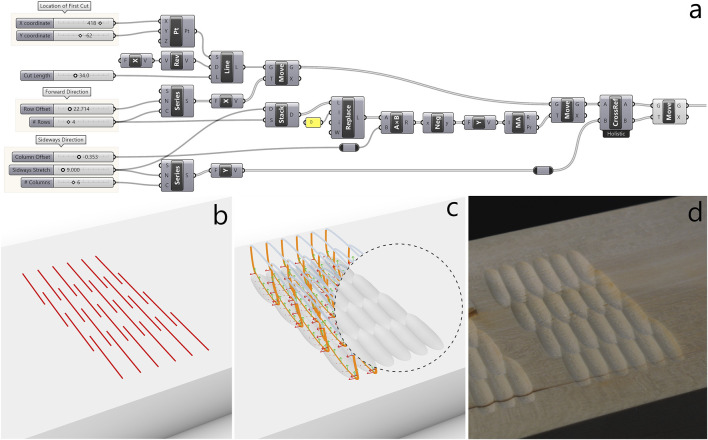
“Naguri pattern” with straight cuts. **(a)** Grasshopper definition of the pattern of straight cuts. **(b)** Pattern of straight cuts used as observations. **(c)** toolpath generated from observations using the ProMP model. Simulated carving result exposed in the circular cut-out. **(d)** Carving result produced by the robot in lime wood.

**FIGURE 8:**
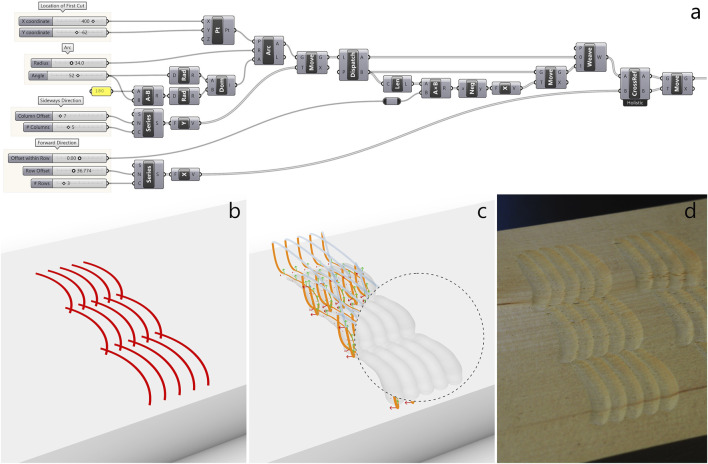
Variation of the “Naguri pattern” with curved cuts. **(a)** Grasshopper definition of the pattern of arcs. **(b)** Pattern of arcs used as observations. **(c)** toolpath generated from observations using the ProMP model. Simulated carving result exposed in the circular cut-out. **(d)** Carving result produced by the robot in lime wood.

“Naguri” is a traditional Japanese surface texturing technique that is among other things applied to beams, wall paneling and furniture. Typically, naguri is a labor-intensive process where gouges or other special tools are used to carve grooves into a wooden surface in regular or random patterns. The resulting surface plays with light and shadow, making it visually dynamic, and is interesting to touch. We created a naguri style texture with straight cuts (Figure 7d) in a regular order and another variation with curved cuts (Figure 8d). Being a regular, repetitive pattern where the arrangement of cuts needs to be finely tuned to make the grooves overlap while remaining clearly defined, the naguri patterns are cases where parametric pattern definitions done in Grasshopper are particularly well-suited (Figures 7a, b). The pattern parameters such as length of cut, numbers and offsets of rows and columns are easily adjusted with number sliders, and the resulting toolpath adapts automatically on any parameter change. The grooves can be simulated by subtracting the volume created by the intersection of the gouge edge with the wood from a model of the wood plank (Figures 7c, 8c). However, this method of simulating cuts does not account for material properties or the physical interaction between the tool and the wood, making it a rather idealized representation. In practice, the carved results came out less pronounced than expected. To account for this, we lowered the XY-plane, where the cuts are planned, below the wood surface by −5.15 mm for the straight naguri pattern and −5.35 mm for the pattern with curved cuts. This adjustment increased the tool’s pressure on the wood, resulting in more pronounced cuts. For further details on the adjustments see Section 4.3. With the adjustments the simulated carvings and the final results match well under visual comparison. A video illustrating the design workflow of the naguri pattern with straight cuts (Supplementary Video 2) and a video of the robot carving the pattern (Supplementary Video 3) can be found in the Supplementary Materials.

#### 4.2.2 Rope

**FIGURE 9:**
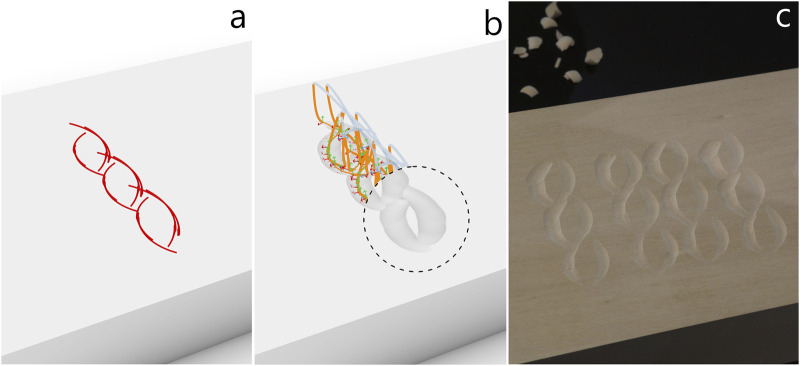
“Rope pattern” made from alternating curved cuts. **(a)** Rope pattern drawn in Rhino using arc and move and copy commands. The drawings are imported into Grasshopper with a geometry pipeline (Figure 5). **(b)** Toolpath generated from observations using the ProMP model. Simulated carving result exposed in the circular cut-out. **(c)** Carving result produced by the robot in lime wood.

Rope-shaped moldings are also traditional decorative elements in wood carving. For creating the twisted rope effect in Figure 9c, one carves a clockwise arc which is continued by an overlapping counterclockwise arc. The next set of arcs is shifted such that it is barely touching the previous set to create a sense of depth. The digital design in this example was drawn manually in Rhino using arc, move and copy commands. Comparing the drawings in Figure 9a and the simulated carving in Figure 9b with the final result in Figure 9c, we see that there are differences between the digital and physical designs. And indeed, the drawing in Figure 9a is the result of an iterative process of doing test cuts and toolpath adjustments to match the desired outcome, rather than an intuitive 2D drawing of the rope. Besides adjusting the drawing in the 2D plane, we steepened the tool angle by applying a constant 4° rotation around the x-axis of the tool frame to make the cuts more pronounced. We observed differences in how the cuts turned out across the pattern, even between identical copies of arcs that were merely placed at different locations in the XY-plane. To address this, we adjusted the location (height) of the XY-plane for the individual cuts in the robot’s base frame, within a range of 4.4–7.1 mm. Otherwise, some of the cuts would come out too deep, eventually making the robot stop, or too shallow and therefore too short, making them differ from the drawing input. The need for such iterative adjustments hints at inaccuracies in our carving pipeline, and potentially in the way we simulate carving in Rhino.

#### 4.2.3 Flower

**FIGURE 10:**
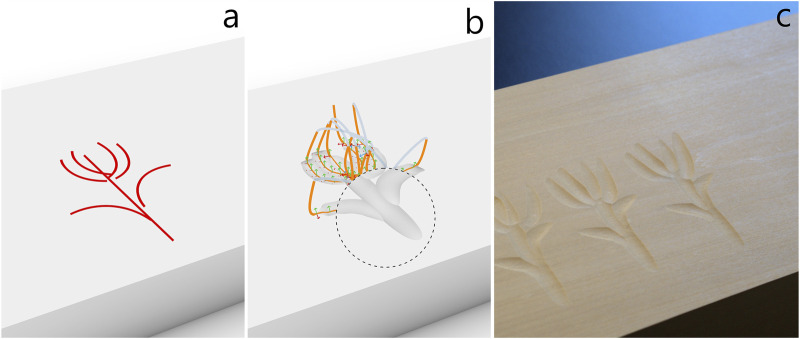
A flower made from straight and curved cuts. **(a)** The flower is manually drawn in Rhino using arc and line commands. The drawings are imported into Grasshopper with a geometry pipeline (Figure 5). **(b)** Toolpath generated from observations using the ProMP model. Simulated carving result exposed in the circular cut-out. **(c)** Carving result produced by the robot in lime wood.

In this example, we carved a flower to demonstrate a less repetitive and more freehand-like design that combines straight and arc-shaped cuts of different directions and radii. Similar to the rope in Section 4.2.2, the flower drawing (Figure 10a) was manually drawn in Rhino and had to be slightly adjusted based on test cuts to get to the desired result. As it can be seen in the drawing, we had to shift the petals slightly to the right with respect to the stem for them to appear centered above the stem in the carved result. To optimize the cuts and make them roughly equally strong marked, we adjusted the location of the XY-plane in the robots base plane in a range from −11.7 mm to 5.2 mm.

### 4.3 Comparison of toolpath adjustments

In the applications in Section 4.2, we had to do slight adjustments to the toolpath to achieve the desired result. For the most part, the adjustments were necessary because the cuts done by the robot were not as pronounced as expected, being too short, narrow and shallow. And when the cuts come out too short and narrow, they can also appear to be misplaced, since they do not align properly or extend to the intended boundaries. To better understand the effect of the adjustments, we conducted a series of straight test cuts with different parameters. We first did a manual cut to have a basic reference. We measure the length of this cut, and aim to recreate it with the robot by drawing a straight line of the same length in Rhino and apply our pipeline. Before executing the toolpaths on the robot, we do two types of adjustments with different intensity: The first adjustment is to lower the location of the XY-plane of the toolpath, which means performing the toolpath slightly beneath the measured height of the wood surface. The second adjustment is to make the tool angle steeper by rotating the tool frame (see Figure 2) slightly around its x-axis in positive direction. To compare the cuts, we record a point cloud of the carved wood with a Zivid One+ 3D camera, which we used to measure the geometries of the cuts. In addition, we looked at how the executed toolpaths compare to the planned toolpaths and what forces and torques act on the tool during execution.

**FIGURE 11:**
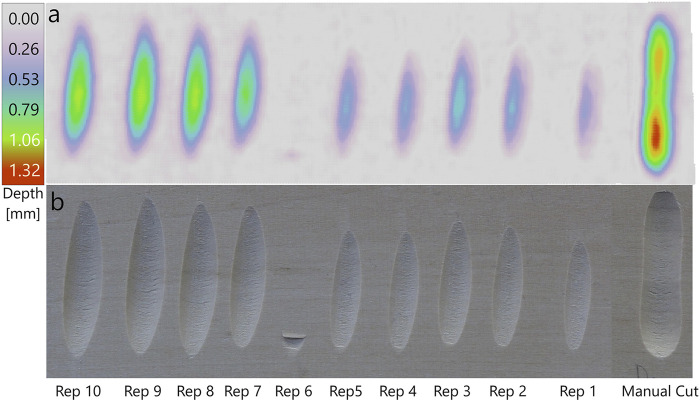
Reproductions of a manual cut with different parameter adjustments. **(a)** Depth map of the cuts generated from a recorded point cloud of the carved wood. **(b)** Photo of the carved wood.

Table 1 shows adjustments and the most relevant dimensions of the different cuts. The manual cut has a length of 36.8 mm and the greatest width, depth and volume. In general, we can only expect the robot’s reproduction of an arbitrary manual cut to match in length but not exactly in width, depth and volume, since we inform our ProMP model only with a 2D line drawing and a cut of similar length can be executed in many different ways. Such a difference in execution is visible in Figure 11 when comparing Rep 1 to Rep 10 - both cuts have roughly the same length, but are shaped differently. The reproductions by the robot come out shorter than the manual cut, even though we aimed for a cut of the same length.

**TABLE 1:** Measurements of the manual and adjusted cuts. “Manual” is the manually done reference cut and Rep 1–10 are the cuts done by the robot. Rep 1 is the reproduction without adjustments, executed in measured XY-plane that was found to be at z=−18.2mm in the robot base frame. Rep 2–6 are the cuts where the XY-plane was lowered below the measured XY-plane. Rep 7–10 are the cuts where the tool angle was steepened. In Rep 10 the same toolpath was executed twice. The XY-plane for Rep 7–10 was at the measured height of −18.2 mm.

Cut	Adjustments		Measurements			
	XY-Plane	Tool Angle	Length	Max. Width	Max. Depth	Volume
	[mm]	[°]	[mm]	[mm]	[mm]	$\left[mm^{3}\right]$
Manual	—	—	36.8	8.9	1.16	159.5
Rep 1	—	—	24.1	5.6	0.41	15.2
Rep 2	−2.8	—	26.2	6.2	0.52	26.2
Rep 3	−3.8	—	27.2	6.9	0.61	35.3
Rep 4	−4.8	—	25.4	6.3	0.48	23.5
Rep 5	−5.8	—	25.5	6.3	0.47	23.7
Rep 6	−6.8	—	—	—	—	—
Rep 7	—	+2	30.8	7.5	0.76	54.1
Rep 8	—	+3	32.8	8.1	0.88	75.9
Rep 9	—	+4	33.9	8.2	0.84	79.9
Rep 10	—	+4	34.0	8.2	0.86	79.2

Rep 10 and 9 come closest to the manual cut in terms of length, but also width, depth and volume. Rep 1, without any adjustments, is the shortest, narrowest, shallowest and has least volume. The appearance of the cuts done by the robot is rather consistent across the adjustments. Lowering the XY-plane seems to make the cuts more pronounced, up to a certain point. While there is a noticeable difference between the unadjusted cut (Rep 1) and Rep 2 and Rep 3, both visually and in primary dimensions, lowering the XY-plane further (Rep 4–6) does not seem to be effective. Rep 6 could not be completed; the robot stopped after a few millimeters cutting (Figure 11b) because the end effector forces exceeded the safety limit.

Steepening the tool angle also makes the cuts more pronounced, and seems to be more effective than lowering the XY-plane. Small positive rotations around the x-axis of the tool frame lead to longer, wider, deeper and more voluminous cuts. In Rep 10, the same toolpath as in Rep 9 was used, but was executed twice to see if a second pass would carve away more wood. However, the differences both in appearance (Figure 11) and measurements (Table 1) are small.

**FIGURE 12:**
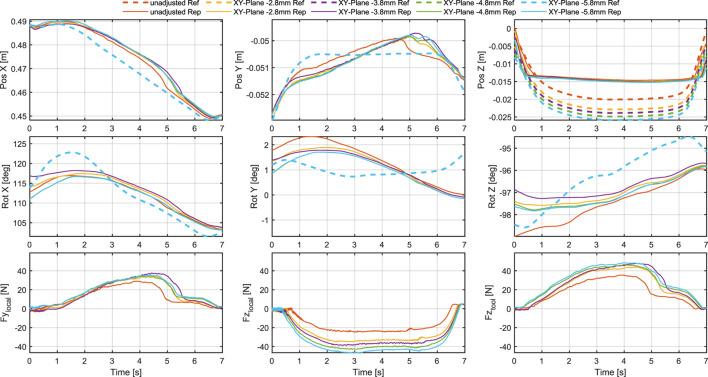
Comparison of tool poses and forces during cuts with different XY-plane adjustments as specified in Table 1. The first row shows the tool position in the robot base frame. The second row shows the tool orientation in Euler angles (ZYX convention) in the robot base frame. The third row shows the forces along the y-axis and z-axis of the local frame of the cuts, and along the z-axis of the tool frame.

**FIGURE 13:**
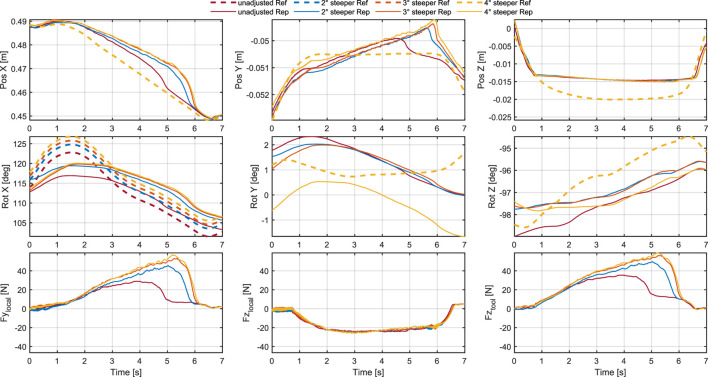
Comparison of tool poses and forces during cuts with different tool angle adjustments as specified in Table 1. The first row shows the tool position in the robot base frame. The second row shows the tool orientation in Euler angles (ZYX convention) in the robot base frame. The third row shows the forces along the y-axis and z-axis of the local frame of the cuts, and along the z-axis of the tool frame.

Looking at the forces acting on the tool in Figures 12, 13 it can be noticed that both lowering the XY-plane and steepening the tool angle increase $Fz_{\mathrm{tool}}$ , i.e., increase the force acting along the tool shaft. However, comparing the forces in the local frame of the cut, $Fy_{\mathrm{local}}$ along the direction of cut and $Fz_{\mathrm{local}}$ normal to the wood surface, we notice that adjusting the tool angle leads to higher force along the cut $Fy_{\mathrm{local}}$ , while the force pressing down on the wood $Fz_{\mathrm{local}}$ remains constant. Lowering the XY-plane leads to higher forces normal to the wood surface and less of an increase of force along the cut.

The observed forces indicate that the reproductions do not lack downward force, but rather have a sub-optimal tool angle. And indeed, the tracking accuracy of the rotations around the x-axis RotX is rather low and the executed tool angle shallower than planned, especially at the beginning of the cut. We repeated the execution of the unadjusted toolpath without any contact with the wood and observed a similar tracking behavior, where the executed tool angle is about 5° shallower than the reference when the cutting is supposed to start. Considering the maximum depth the tool reaches in PosZ in Figures 12, 13, we notice that the tool makes contact with the wood in a higher region on the z-axis than we expected based on our measurements in the setup. This means that there have to be inaccuracies in our setup. For example, in our measurement of the wood surface, the calibration of the tool frame in the flange frame of the robot, or somewhere else in the robot. As a result, the reproductions, including the unadjusted one, were executed slightly below the measured height of the wood surface, increasing the downward force $\left(Fz_{\mathrm{local}}\right)$ of the tool on the wood. This also means the tool made contact with the wood, and potentially began cutting, before reaching the planned position and orientation, resulting in unintended deviations in the process.

## 5 Discussion

The applications presented in Section 4.2 demonstrate that our framework can be used for carving applications. However, there are several issues that need further investigation and improvement. Although the approach in general is functional, the resulting toolpaths require adjustments to match the desired output in wood carving. Possible reasons include too few or bad demonstrations, bad modeling of the data, unsuitable robot and controller or inaccuracies in TCP calibration.

We observed throughout the experiments that the cuts come out less marked than expected when the toolpaths generated by our approach are executed by the robot. However, with slight adjustments to the toolpaths, the cuts come close to the intended outcome. For the carving applications in Section 4.2, we gave an offset on the XY-plane in which the toolpaths are planned, such that it lays lower than the wood surface measured in the robot’s base frame and/or adjusted the tool angle, such that the tool cuts into the wood at an steeper angle. Both of these corrections lead to more marked cuts, but it appears from the comparison experiment in Section 4.3 that, in our case, steepening the tool angle is the preferable option since it leads to higher forces in direction of the tool shaft rather than higher forces normal to the wood surface. We presume that loading the tool axis is a better use of the forces available at the robot’s end effector since it increases the pressure on the cutting edge, leading to more pronounced cutting without overloading the robot. However, a more in-depth investigation of the cutting mechanics of handheld wood carving tools would be necessary to provide more definitive answers.

In this paper, we assessed the generated toolpaths based on the quality of the wood carving results. We have not done a separate validation of the learned ProMP model and can therefore not fully rule out that our model produces suboptimal toolpaths that make the adjustments necessary. There are multiple factors during learning and application that can affect the quality of trained model and carving results, such as the number and quality of the demonstrations and their alignment, the number of observations that each drawing input is discretized into and the hyperparameters of the BGMM. With a relatively small set of demonstration data used in this paper, the generalization capabilities of the model will be limited. We noticed that if we drew curves that were far away from the demonstrated cuts, the toolpaths degenerated by, for example, tilting too much to one side. However, since we trained a small model representing only a few skills and we did the demonstrations ourself, we could gain some intuition for the range of drawings we could use in our applications based on visual inspection and robot simulation of the toolpaths in Rhino. The toolpaths generated for the applications in Section 4.2 appeared plausible, and we suspect that the robot and controller we used have a rather large impact on the quality of the carving results and may be the main cause for the adjustments.

Considering that we use a low payload collaborative robot which runs a task-space impedance controller, we cannot expect the trajectory tracking performance of a calibrated industrial robot, especially when interacting with a relatively stiff material such as wood. This is one possible explanation why the cuts come out less marked than expected when we do not apply any adjustments. The impedance controller does not build up enough force to perform the full cut that, without any adjustments, only leads to small deviations from the reference path. Lowering the XY-plane pre-loads the tool, such that the small deviations evoked by the contact between wood and tool lead to enough force to cut the wood.

Steepening the tool angle *per se* does not pre-load the tool as lowering the XY-plane does, but it causes the cutting edge to more easily catch in the wood, which, over the course of the execution, leads to an increasing deviation from the reference toolpath and results in a stronger cutting force. Considering the low tracking performance of rotations around the x-axis of the tool, which results in a shallower tool angle than planned, manually steepening the tool angle acts less as a modification and more as a correction that brings the executed movement closer to the toolpath planned by our pipeline.

When creating the rope (Section 4.2.2) and flower (Section 4.2.3), we had to draw some of the cuts at counterintuitive locations in the XY-plane, making the drawings look slightly distorted. For example, the petals of the flower had to be shifted slightly to the right of the stem in the drawing, and the transitions where the arcs change direction in the rope had to have a gap between even though the pattern should be continuous there. Such modifications were necessary because the cuts carved into the wood ended up at slightly different locations than in the drawing, thus not exactly matching the desired design. We have no definitive answer to why modifications in the XY-plane are necessary, but we suspect that the precision and strength of the overall robot setup is just not sufficient. We assume that as soon as the robot comes in contact with the wood, the tracking accuracy declines and the shape and length of cuts change. Also, as we noticed in Section 4.3, the boundaries of the cuts change substantially when the robot does not manage to cut as deep as intended. And while our adjustments help to make more fully formed cuts, they could lead to misalignment in the XY-plane since they may change the point of the toolpath at which the tool touches the wood and the cut starts. For example, when lowering the XY-plane, the tool will touch the wood earlier, during the approach movement, and since the approach is not necessarily a straight downward move, the starting position of the cut changes.

Another hint at the setup as a major source of imprecision is that we had to use different correction parameters for identical cuts at different locations. In the rope, for instance, we had to use other XY-plane offsets for the cuts at the end and the beginning, even though the toolpaths for the cuts are identical, just placed at different locations on the wood. This shows that it is at least not only shortcomings of the carving skills represented by our model that require adjustments.

In general, it is unfavorable that we had to adjust the toolpaths generated by our pipeline further to achieve the desired carving results with our robot setup, but at the same time it demonstrated an important quality of our approach from a fabrication/user interface perspective: Our integration of ProMPs in Rhino/Grasshopper enables iterative design workflows and rapid prototyping with toolpaths generated from human motor skills. In future applications, the user should ideally not have to iterate over adjustments to make the toolpaths work but rather iterate over their design - to experiment, refine and generate variation.

We could show that our approach, on a basic level, provides CAM-like functionality by converting a digital design into machine instructions based on human craft skills. The chosen trajectory-based skill representation enables the teaching of cuts that follow arbitrary, uniquely shaped paths while capturing correlations between the path shape and other parameters, such as tool orientation. In contrast, adopting a shape-agnostic representation, such as the one used for straight cuts in Brugnaro and Hanna (2017), results in the loss of relationships between path shape and other parameters. For example, such a representation assumes that the tool angle with respect to the wood surface at 80% of a straight cut is identical to the angle at 80% of an s-shaped cut. At the same time, a shape-agnostic approach is a more general skill representation that has the potential to offer greater design freedom without having to demonstrate cuts for all geometries the maker wants to use in their digital design. Our current approach does not generalize well over large variations in the shapes of cuts, which limits the maker in the design process to geometries that are relatively similar to the provided demonstrations. As a result, our approach falls short of being equivalent to traditional CAM tools, where it is expected to be able to generate machine instructions for all geometries that are technically possible to make with the selected tools and machines. Demonstrating cuts for all possible geometries is impractical. Therefore, the proposed system is better suited for computing machine instructions for specific, tailored/maker-defined manufacturing operations (e.g., carving unique decorative elements) rather than for producing diverse 3D objects from a block of material, as is typical in CAM for milling.

In conclusion, we have demonstrated how LfD can be utilized in digital fabrication through an integration of movement primitives in CAD software. Our approach enables users to program toolpaths corresponding to intricate human craft skills by defining geometries in a CAD environment. However, for the carving applications in this paper, some manual adjustments to the toolpaths were necessary, indicating that the model’s generalization is still limited and highly dependent on calibration and user expertise. Our approach functions in principle similar to a CAM tool but where the user can add new machining/manufacturing operations by demonstrating the operation to the robot. Additionally, the user interface seems to be suitable for supporting an iterative workflow.

However, the presented approach is not yet ready for most real world applications. We consider future work in two directions: First, in the context of robot wood carving, we plan to investigate alternative representations for carving skills. Is carving technique inherently tied to the shape of cut, or is it possible to find a more general, underlying representation that can be utilized for a diverse range of cuts? In addition, how can the change in geometry of a workpiece caused by a cut be modeled effectively for use in machine learning algorithms? Furthermore, we aim to incorporate other task parameters in addition to the tool pose into the learned model to make the learned skills more adaptive to the anisotropic properties of wood. The direction of the grain with respect to the cut could be a relevant factor, but also the wrenches at the tool tip present during demonstration and reproduction. Measuring the tool wrenches during kinesthetic teaching is not straightforward and requires modifications to the conventional hand tools used in this paper. However, including the wrenches in the model would allow for hybrid force-position control strategies which we deem more appropriate than our current implementation of an impedance controller that we consider as a major weakness of our results. Additionally, to address the inaccuracies observed in our experiments, we plan to use an industrial robot arm for future reproductions of wood carving cuts, ensuring greater consistency and more conclusive findings. And second, in the context of a hybrid LfD-CAM interface, we intend to do an in-depth evaluation of our approach of using LfD in manufacturing by conducting a user study involving hobbyists or professionals and perhaps other manufacturing techniques like painting or clay molding.

## Data Availability

The raw data supporting the conclusions of this article will be made available by the authors, without undue reservation.
